# Cation Substitution in Earth‐Abundant Kesterite Photovoltaic Materials

**DOI:** 10.1002/advs.201700744

**Published:** 2018-01-29

**Authors:** Jianjun Li, Dongxiao Wang, Xiuling Li, Yu Zeng, Yi Zhang

**Affiliations:** ^1^ Institute of Photoelectronic Thin Film Devices and Technology and Key Laboratory of Photoelectronic Thin Film Devices and Technology Tianjin Nankai University Tianjin 300071 China; ^2^ Institute of New Energy Technology Jinan University Guangzhou 510632 China

**Keywords:** band bending, cation substitution, graded bandgaps, kesterite solar cells, open‐circuit voltage deficit

## Abstract

As a promising candidate for low‐cost and environmentally friendly thin‐film photovoltaics, the emerging kesterite‐based Cu_2_ZnSn(S,Se)_4_ (CZTSSe) solar cells have experienced rapid advances over the past decade. However, the record efficiency of CZTSSe solar cells (12.6%) is still significantly lower than those of its predecessors Cu(In,Ga)Se_2_ (CIGS) and CdTe thin‐film solar cells. This record has remained for several years. The main obstacle for this stagnation is unanimously attributed to the large open‐circuit voltage (*V*_OC_) deficit. In addition to cation disordering and the associated band tailing, unpassivated interface defects and undesirable energy band alignment are two other culprits that account for the large *V*_OC_ deficit in kesterite solar cells. To capture the great potential of kesterite solar cells as prospective earth‐abundant photovoltaic technology, current research focuses on cation substitution for CZTSSe‐based materials. The aim here is to examine recent efforts to overcome the *V*_OC_ limit of kesterite solar cells by cation substitution and to further illuminate several emerging prospective strategies, including: i) suppressing the cation disordering by distant isoelectronic cation substitution, ii) optimizing the junction band alignment and constructing a graded bandgap in absorber, and iii) engineering the interface defects and enhancing the junction band bending.

## 1 Introduction

Concerning the rapid increase of global energy demand and the growing severe environmental issues caused by the consumption of traditional fossil fuels, clean, safe, and renewable energy sources are in urgent need. Photovoltaic (PV) technology that can directly delivers the inexhaustible solar energy to clean electricity is considered to be an attractive solution.^1^ Taking advantages of less source material cost and adapted to flexible substrates,^2^ thin film PV technology is an important branch to make solar electricity more cost‐effective and prevalent in various application situations, namely building integrated photovoltaics (BIPV), unmanned aircraft systems,^3^ wearable power supply, and so on.^4^ Cu(In,Ga)Se_2_ (CIGS) and CdTe thin‐film solar cells have demonstrated over 20% power conversion efficiency (PCE).^5^, ^6^, ^7^, ^8^, ^9^, ^10^ However, the scarcity of In and Te elements and environmentally hazardous Cd constrain the large‐scale commercialization and the reduction in production costs.^11^, ^12^, ^13^, ^14^ In the last decade, the new emerging thin‐film photovoltaic devices based on kesterite structural semiconductors Cu_2_ZnSn(S, Se)_4_ (CZTSSe) have attracted considerable attention, owing to its large potential as a candidate for high‐performance photovoltaic technology with earth‐abundant source materials.^11^, ^12^, ^15^, ^16^, ^17^ Inheriting the device structure from the predecessor chalcopyrite CIGS solar cells,^18^, ^19^ and benefitting from the advanced first‐principle material calculations which give clear directions of chemical potential window, energy band, and defects of kesterite material family,^11^, ^20^, ^21^, ^22^ CZTSSe thin‐film solar cells have experienced a significant increase in power conversion efficiency from about 5% in 2004 to the record 12.6%.^15^ However, the current record efficiency of CZTSSe devices has been pinned at 12.6% for four years. In other word, the development of CZTSSe solar cells is experiencing a stagnation. However, this kind of stagnation is not rare in the 40 years’ history of well‐developed CIGS and CdTe thin‐film solar cells.^23^

**Figure 1:**
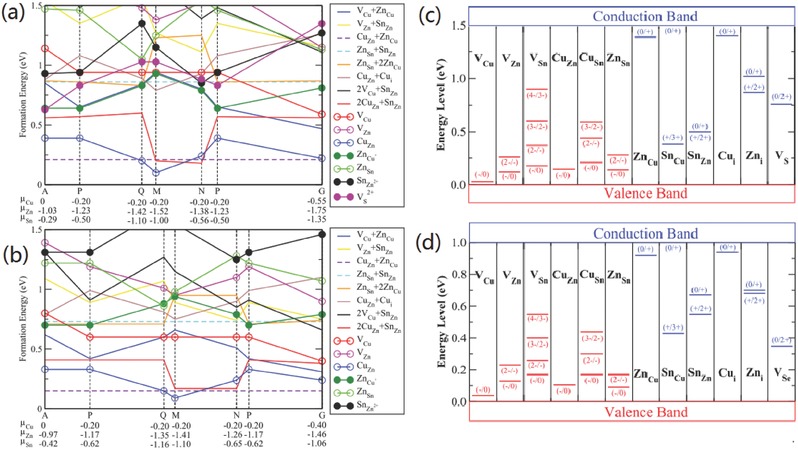
a) The formation energy of low‐energy defects in Cu_2_ZnSnS_4_. b) The formation energy of low‐energy defects in Cu_2_ZnSnSe_4_. The Fermi energy is assumed at the top of the valence band (p‐type conditions), and thus the donor defects are fully ionized. c) The ionization levels of intrinsic defects in the bandgaps of Cu_2_ZnSnS_4_. d)The ionization levels of intrinsic defects in the bandgaps of Cu_2_ZnSnSe_4_ (bottom). The red bars show the acceptor levels and the blue bars show the donor levels, with the initial and final charge states labeled in parentheses. The calculated (using density functional theory) bandgaps are corrected to the experimental values of 1.5 and 1.0 eV, respectively. Reproduced with permission.^21^ Copyright 2013, Wiley‐VCH.

**Figure 2:**
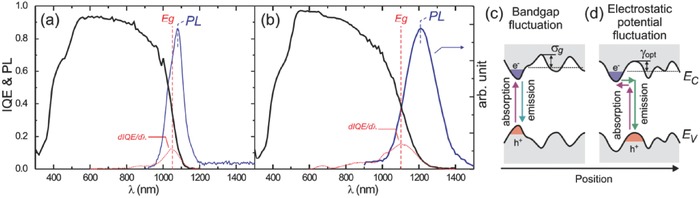
The internal quantum efficiency, bandgap as determined from the IQE inflection point and the photoluminescence spectra (taken using a 532 nm solid state laser) of high performance. a) CIGSSe (*E*_g_ = 1.19 eV) and b) CZTSSe (*E*_g_ = 1.13 eV) devices. c) Schematic of bandgap fluctuations and d) electrostatic potential fluctuations. Reproduced with permission.^24^ Copyright 2013, AIP Publishing LLC.

The current bottleneck that CZTSSe solar cell encounters is the large *V*_OC_ deficit (*E*_g_/q‐*V*_OC_), reported unanimously by various literature.^16^, ^24^, ^25^, ^26^, ^27^ Even the CZTSSe solar cells with the best performance suffer from the low *V*_OC_ that never better than 60% of the *V*_oc max,_ which is expected from the Shockley–Queisser radiative limit (S–Q limit),^28^ as reviewed by Bourdais et al.^25^ Recent years, considerable studies have been conducted to disclose the mechanism of the large *V*_OC_ deficit. These results show that the large *V*_OC_ loss of CZTSSe device can be attributed to various reasons, of which the most accepted ones are abundant point defects and defect clusters (i.e., cation disordering),^17^, ^24^, ^25^ and associated severe band tailing,^29^, ^30^, ^31^ band fluctuations caused by microinhomogeneities in composition and anion substitution,^32^, ^33^ as well as unpassivated junction interfaces and undesirable conduction band offset (CBO).^15^ Among these issues, the abundant point defects, defect clusters, and associated band tailing attract the most attention and are extensively studied recently. As shown in **Figure** **1**a,b, Cu and Zn atoms can substitute with each other with low enthalpy cost because of the close cation sizes and small chemical mismatch of Cu^+^ and Zn^2+^ in kesterite system.^21^, ^34^ Therefore, a large population of antisite defects such as Cu_Zn_ and Zn_Cu_, and related defects complexes are prevalent in the kesterite structure.^21^ As a consequence, severe electrostatic potential fluctuation and associated band tailing are introduced, as evidenced by the pronounced PL red‐shifting (**Figure** **2**).^24^, ^35^ In addition, the high concentration of acceptor‐like Cu_Zn_ defects will pin the interface Fermi level to a low energy level,^2^ thus reducing the band bending in the absorber and weakening the electric field of the heterojunction interfaces. As a result, both *V*_OC_ and fill factor dramatically deteriorate.

Considering the above‐mentioned issues of CZTSSe materials and learning from the successful CIGS solar cells, some prospective strategies to break through the current *V*_OC_ limit of CZTSSe devices are proposed: (i) suppressing the Cu/Zn cation disordering and associated band tailing by distant isoelectronic cation substitution; (ii) optimizing the junction interface conduction band offset and engineering a bandgap grading similar to CIGS thin film solar cells; (iii) constructing a buried homojunction and thus enhancing the band bending in absorber by surface type inversion. Proper isoelectronic cation substitution in the host CZTSSe structure is one of the most promising methods to realize all the above goals. In past years, lots of efforts have been paid to find out an effective cation substitution method to address particular issues.^36^, ^37^, ^38^, ^39^, ^40^, ^41^, ^42^ Therefore, a comprehensive analysis among the various cation substitute methods is needed to tease out key technologies to overcome the current obstacles for kesterite solar cells and further advance the efficiency roadmap of this photovoltaic technology.

In this review, our concerns focus on the strategies to overcome the large *V*_OC_ deficit of kesterite thin‐film solar cells by proper cation substitution technology, including suppressing the cation disorder by distant cation substitution, tuning the structure of bandgap by engineering uniform and graded cation substitution, and engineering the junction interface defects to enhance the band bending in absorber. Meanwhile, we also review the current state‐of‐the‐art researches on the cation substitution for kesterite thin‐film solar cells.

## 2 Suppressing the Cu/Zn Disorder by Distant Cation Substitution

The multinary feature of CZTSSe system introduces a large possibility of abundant point defects in these materials.^11^ As shown in Figure 1, predicted by the density functional theory (DFT) calculation, various point defects including antisite defects and vacancies may exist in the CZTSSe system. In addition, the formation energy of the charge‐compensated defect complexes is expected to be smaller than that of individual defects because of the charge transfer and attractive Coulomb interaction between positive and negative charged defects.^21^ For instance, the formation energy of Cu_Zn_+Zn_Cu_ clusters is particularly low, which makes these defect clusters rather prevalent in CZTSSe materials. By forming charge‐compensated defect clusters, the point defect density could be reduced effectively. However, this self‐compensation effect also induces an adverse factor: the formation energy of 2Cu_Zn_+Sn_Zn_ clusters is quite small and these clusters can be in high concentration even in stoichiometric samples with Cu/(Zn+Sn) and Zn/Sn ratios near 1, though the formation energy of individual deep defects Sn_Zn_ is relatively large. As a result, high concentration deep trap states are introduced and the electronic band gap is reduced.^43^

The constitute of point defects and defect clusters depends on the specific stoichiometric composition of the film.^11^, ^21^ To avoid the detrimental deep defects Cu_Sn_, Sn_Cu_, Sn_Zn_, and suppress the concentration of deep trap states 2Cu_Zn_+Sn_Zn_, the general composition targeted for photovoltaic devices is Cu‐poor and Zn‐rich (e.g., Cu/(Zn+Sn) ≈ 0.8 and Zn/Sn = 1.0–1.1), which has been demonstrated as a proper constitution by current best performance CZTSSe devices.^15^, ^18^, ^44^, ^45^ Despite the competitive Zn(S,Se) phase is easy to form in this narrow phase stability zone,^46^, ^47^ Cu_Zn_ antisite defects become the dominant point defects because they cost the lowest enthalpy, thus endowing the p‐type conductivity to typical CZTSSe materials applied for photovoltaics.^11^, ^21^, ^47^ Unfortunately, as shown in Figure 1c,d, Cu_Zn_ antisite is not a shallow defect (about 100–200 meV above the valence band maximum, VBM) that comparable to V_Cu_ (about 20 meV above VBM) which acts as the dominant acceptor and gives the excellent electronic properties of CIGS materials.^2^, ^21^ The lower formation energy of Cu_Zn_ antisite defects in CZTSSe than V_Cu_ vacancy over all the chemical potential makes CZTSSe difficult to get rid of the undesirable Cu_Zn_ defects. Our previous results of admittance measurements show that the density of V_Cu_ can reach to a level (10^16^ cm^−3^) close to the dominant acceptor Cu_Zn_ in CZTSe under Cu‐poor and Zn‐rich condition,^45^ indicating the formation energies of V_Cu_ and Cu_Zn_ may be close in pure selenide CZTSe with Cu‐poor and Zn‐rich composition. This could be a part of the explanation why CZTSSe solar cells with higher Se content usually present less *V*_OC_ deficit and higher performance than those with high sulfur content.^48^, ^49^

Recent efforts aimed at the cation antisite disordering of CZTSSe reveal that the state of ordering and disordering of CZTSSe materials can transform to each other, which strongly depends on postannealing conditions, such as temperature, dwelling time, and cooling rate.^50^, ^51^, ^52^ The results of the study by Rey et al. show that the transformation temperature of ordering and disordering is near 200 °C.^51^ By long and low temperature postdeposition annealing at 75 to 150 °C, the disordering and associated band tailing can be reduced as demonstrated by the pronounced increase of optical bandgap by about 100 meV.^24^ However, despite that the long‐time annealing treatment (e.g., from several hours to one day) is less feasible in practice, the reduced disordering of CZTSSe is not translated to substantial *V*_oc_ improvement. More in‐depth research is needed to interpret the reason why the severe *V*_OC_ deficit still exists.

Since the Cu/Zn disordering mainly originates from the close ionic radius and chemical electronic properties of Cu^+^ and Zn^2+^, a more prospective strategy to avoid the Cu/Zn disordering is introducing larger ionic radius mismatch by substantially larger or smaller cation substitution for Cu^+^ or Zn^2+^. Concerning the structural stability after the cation substitution, Ag for Cu substitution, and Ba for Zn substitution are two promising solutions to avoid the disordering in the lattice,^2^ despite Ba substitution may lead to crystal structure changes.^53^ In this section, recent studies on cation substitution (including Ag, Cd, Ba, and other transition metal elements) and their effects on the cation disordering are discussed.

### 2.1 Ag Substitution for Cu

**Figure 3:**
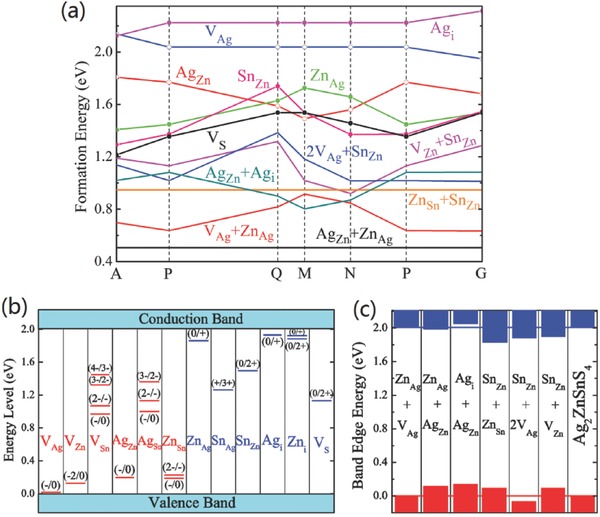
a) The calculated formation energy change as a function of the elemental chemical potentials (growth conditions) for low‐energy defects and defect complexes in Ag_2_ZnSnS_4_. b) The transition energy levels of intrinsic point defects in the bandgap of Ag_2_ZnSnS_4_ and c) the band edge shift caused by the low‐energy defect complexes. The GGA/DFT gap is corrected to the experimental value of 2.01 eV. The band edge shift is calculated assuming one defect complex in a 64‐atom super‐cell. Reproduced with permission.^55^ Copyright 2015, Wiley‐VCH.

The radius of Ag^+^ (1.14 Å) is substantially larger than that of Cu^+^ (0.74 Å) and Zn^2+^ (0.74 Å), making it a favorable option to substitute Cu by Ag.^14^ Theoretically, the DFT calculation for Ag_2_ZnSnS_4_ (AZTS) models by Chen and Chagarov et al. has predicted accordant encouraging electronic properties of this system.^39^, ^54^, ^55^ Not only attributed to the substantially larger ionic size of Ag than that of both Cu and Zn, but also on account of the much lower valence band edge (by 0.74 eV) of AZTS than that of CZTS, the formation energy of Ag_Zn_ antisite in Ag_2_ZnSnS_4_ (ZATS) is significantly larger than that of Cu_Zn_ antisite in CZTS, as well as the formation energy of related defect complexes such as Ag_Zn_+Zn_Ag_ and 2Ag_Sn_+Sn_Zn_ in AZTS (**Figure** **3**a).^55^Consequently, the concentration of intrinsic defects in AZTS is expected to be an order (or more) magnitude lower than that in CZTS.^55^ Moreover, the Ag‐ and Zn‐related defects transition energy levels are shallower, and the corresponding band edge shifting caused by related defect clusters is smaller than that of CZTS (Figure 3b,c). Therefore, Ag for Cu substitution in the CZTSSe system is expected to effectively suppress the cation disordering and associated band tailing.

**Figure 4:**
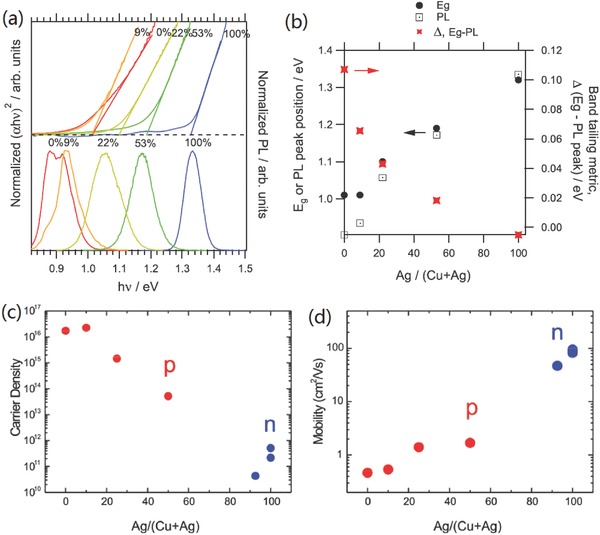
a) Normalized Tauc plots created from UV–vis measurements (top) and normalized PL spectra (bottom) as a function of Ag/(Ag + Cu), b) overlay of extracted *E*_g_ values (filled circles) and room‐temperature PL peak positions (open squares) as a function of Ag/(Ag + Cu) in ACZTSe. The energetic difference between the room‐temperature bandgap and PL peak position is overlaid in red xs. c) Carrier density, d) majority carrier mobility of Ag/(Ag + Cu) ratio. Reproduced with permission.^57^ Copyright 2016, Wiley‐VCH.

**Figure 5:**
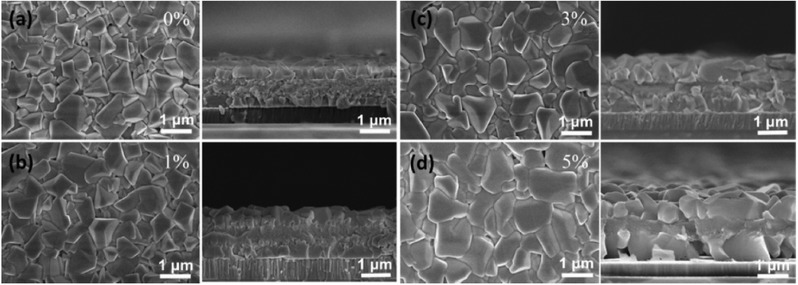
Top‐view and cross‐sectional SEM images of the selenized (Cu_1−_ *_x_*Ag*_x_*)_2_ZnSn(S,Se)_4_ thin films with different ratios of Ag/(Ag + Cu): a) 0, b) 1, c) 3, and d) 5%. Reproduced with permission.^38^ Copyright 2017, American Chemical Society.

Inspired by the above‐mentioned encouraging theoretical results, increasing attention has been addressed to experimental research on AZTS, AZTSe, and their alloys ACZTSSe, as light absorbers for photovoltaics. Gong et al. have performed a systematic research on the crystallographic and optical properties of (Cu,Ag)_2_ZnSnS_4_ (CAZTS) and (Cu,Ag)_2_ZnSnSe_4_ (CAZTSe) solid solutions with a varied ratio of Ag/(Ag+Cu).^56^ They confirmed that both types of CAZTS and CAZTSe solid solutions have a tetragonal kesterite‐type structure by crystal structure refinement.^56^ The bandgap of CAZTS and CAZTSe is continuously tunable from 1.5 to 2.01 eV and from 1.0 to 1.34 eV, respectively, both with a small bowing.^56^ Owing to more suitable bandgap for solar spectrum and better electronic properties, (Cu,Ag)_2_ZnSn(S,Se)_4_ (CAZTSSe) with high Se content is extensively investigated as absorber film for photovoltaics in past two years. Gershon et al. reported that the extent of band tailing in CAZTSe decreases apparently with the increase of Ag content, as evidenced by the significant decrease of the difference between room temperature photoluminescence (PL) peak position and the optical bandgap energy (**Figure** **4**a,b).^57^ The difference is decreases from 110 meV (for pure CZTSe) to 0 meV (for pure AZTSe), which verifies the results of theoretical calculation by Chen and Chagarov et al.^54^, ^55^ As a consequence, with 10% Ag substitution, the *V*_OC_ deficit is reduced from about 660 to 580 mV, and the device performance is improved significantly to 10.2% efficiency using coevaporation process.^57^ Similar results are also found by Wu et al. using solution‐process approaches. Their results indicate that the structural and electronic properties of Ag‐doped CAZTSSe are more sensitive to the content of Ag incorporation.^38^ Only 1% to 5% additive of Ag in precursor solution makes a pronounced difference in crystallinity and electronic properties, as shown in **Figure** **5**. In accordance with the calculated results, they found the free carrier density and defects energy level decreases with the increase of Ag incorporation from 0% to 3%, while more Ag incorporation (5%) deteriorates the electronic properties, which indicates an optimal extent of Ag substitution in the p‐type CAZTSe system may exist. With suitable Ag doping, the *V*_OC_ deficit is significantly reduced from 727 mV (CZTSSe without Ag) to 601 mV (CAZTSe with 3% Ag), which gives rise to the boost of device performance from 7.39% to 10.36%. Reduced band tailing and associated *V*_oc_ deficit by appropriate Ag incorporation in CZTSSe are also found in other researches.^58^, ^59^, ^60^ These encouraging results indicate that Ag substitution may be a promising method to overcome the cation disordering and associated *V*_oc_ limitation of kesterite base solar cells by incorporated with state‐of‐the‐art manufacture technology.

### 2.2 Cd Substitution for Zn

Cu_2_CdSnS_4_ (CCTS) is a p‐type semiconductor similar to CZTS, with tetragonal stannite structure and suitable bandgap (about 1.4 eV) matching well with solar spectrum.^61^ The results of first‐principle calculation by Wei and co‐workers show that CCTS is thermodynamically stable in a narrow chemical potential region,^39^ which is verified by various experimental results.^37^, ^58^, ^59^, ^60^, ^61^, ^62^ These characteristics of CCTS indicate that Cd‐alloyed Cu_2_(Zn,Cd)Sn(S,Se)_4_ (CZCTSSe) materials also possess large potential as a photovoltaic absorber. Though Cd is highly toxic and could pose an environmental risk, Cd‐incorporation in kesterite still attracts considerable attention.

**Figure 6:**
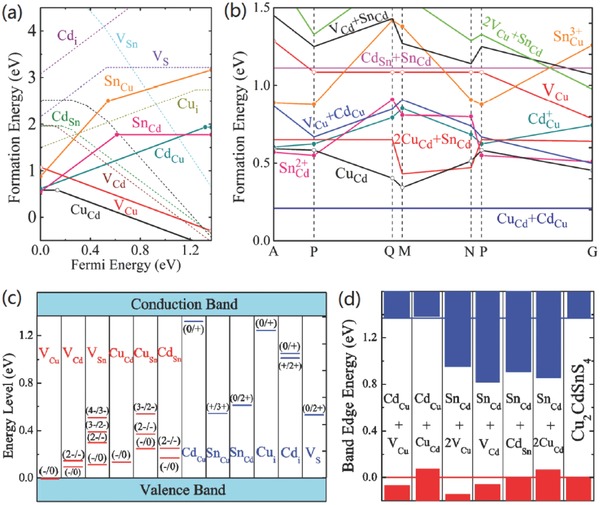
The calculated formation energy change of defects in Cu_2_CdSnS_4_ a) as a function of the Fermi level at the chemical potential point P and b) as a function of the elemental chemical potentials. The Fermi level is assumed to be at the VBM (p‐type conditions), and therefore, all donor defects are ionized in panel (b). c) The transition energy levels of intrinsic point defects in the bandgap of Cu_2_CdSnS_4_, and d) the band edge shift caused by the low‐energy defect complexes. The GGA/DFT bandgap is corrected to the experimental value of 1.38 eV. The band edge shift is calculated assuming one defect complex in a 64‐atom super‐cell. Reproduced with permission.^55^ Copyright 2015, Wiley‐VCH.

Cd (0.92 Å) with larger ionic radius than that of Cu and Zn is expected to be a favorable isoelectronic substitution for Zn, since the larger size mismatch between Cu and Cd is expected to improve the formation energy of Cu_Cd_ and Cd_Cu_ antisites. However, the results of DTF calculation performed on Cu_2_CdSnS_4_ (CCTS) system is in contrast with this expectation.^55^ As shown in **Figure** **6**a,b, the calculated formation energy of Cu_Cd_ is still the lowest‐energy acceptor defects in CCTS in any chemical potential condition, and just slightly higher than that of Cu_Zn_ (by about 0.2 eV). So the formation energy of Cu_Cd_+Cd_Cu_ defect complex is also low, as well as that of 2Cu_Sn_+Sn_Cd_ defect complex.^55^ Even worse, the dominant defects (Cu_Cd_) transition energy level is deep and the band edge shifting caused by related defects complexes is also as large as that of CZTS (Figure 6cd). Therefore, theoretically, Cd substitution for Zn site is not an effective method to avoid the cation disordering and associated band tailing.

In experimental, Pilvet et al. first observed that there is little difference of PL peak red shifting between CZTS (0.25 eV) and CCTS (0.26 eV),^61^ which indicates the issue of band tailing remains unsolved. Even so, recent studies on Cd‐substitution give a consilient conclusion that suitable Cd‐incorporation is of pronounced benefit to improve the device performance of kesterite thin‐film solar cells.

**Figure 7:**
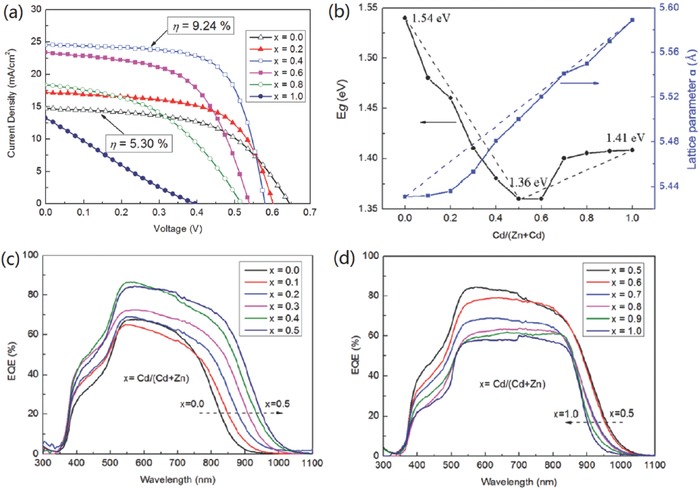
a) *J–V* characteristics of Cu_2_Zn_1−_ *_x_*Cd*_x_*SnS_4_ thin film solar cells (*x* = 0–1.0). b) The dependence of bandgaps (left) and lattice parameter a (right) with the ratio of Cd/(Zn+Cd) for Cu_2_Zn_1−_ *_x_*Cd*_x_*SnS_4_ thin films. EQE of Cu_2_Zn_1−_ *_x_*Cd*_x_*SnS_4_ thin film solar cells with different *x*: c) *x* = 0.0–0.5 and d) *x* = 0.5–1.0. Reproduced with permission.^37^ Copyright 2015, Wiley‐VCH.

By fine adjusting the ratio of Zn/Cd using sol–gel method, Su et al. significantly improve the device performance from 5.3% to 9.24% with 40% Cd substitution (**Figure** **7**a).^37^ Higher substitution amount of Cd is found to dramatically deteriorate the device performance. Though the issue of band tailing remains unsolved in the high‐performance CZCTS solar cells as indicated by the relatively slow EQE decay below *E*_g_ (Figure 7c), the *V*_OC_ deficit is reduced by more than 100 mV compared to the CZTS device.^37^ Besides of the possible modification of conduction band offset of the junction interface by tuning the absorber bandgap (will be discussed in Section 3), the increase of device performance more likely benefits from the improvement of crystalline size and associated less grain boundary defects, and the reduction of secondary phases, such as ZnS. They observed that the average grain sizes of CZCTS thin films increase considerably as Cd‐substitution increases from 0 to 40%. In contrast, the crystallinity decreases when the ratio of Cd/(Zn+Cd) is above 0.5.^37^ Further investigation shows that large amount substitution of Zn by Cd leads to phase transformation from kesterite to stannite when Cd/(Zn+Cd)>0.6. Another interesting result observed by Su et al. is the decrease of carrier density and related increase of depletion region width when Cd/(Zn+Cd)>0.5.^37^ The collapse of device performance in this substitution level may be attributed to the poor crystallinity, low conductivity, and possible high density of deep recombination centers which is indicated by the low EQE response at whole absorption spectrum of corresponding devices (Figure 7d).

Recently, Yan et al. fabricated 11.5% efficiency Cd‐doped Kesterite Cu_2_Zn*_x_*Cd_1−_ *_x_*SnS_4_ solar cells by sulfurizing chemical bath‐deposited CdS on top of cosputtered Cu/ZnS/SnS precursors.^62^ In their research, Urbach tail energy (*E*_Urbach_) model is used to estimate the band tailing. The lower *E*_Urbach_ value indicates a more clean band edge with less band tailing (i.e., better lattice ordering in the absorber material). Photothermal deflection spectroscopy (PDS), which is a direct measurement of the optical absorption and very sensitive to subband absorption, was used to determine the *E*_Urbach_. The PDS measurements were performed on CZTSSe and CZCTS films on glass substrates to exclude the interference of the Mo back contact absorption. The derived *E*_Urbach_ of CZTS and CZCTS are 65 and 45 meV, respectively, indicating a reduction in band tailing of CZTS absorber through appropriate Cd substitution. These values are much larger than those *E*_Urbach_ (13–31 meV) obtained by Miller et al. who used transient photocapacitance measurements to characterize the *E*_Urbach_.^63^ The difference may originate from the employed measurement methods. Time‐resolved photoluminescence (TRPL) measured minority carrier lifetime values of Cd‐doped CZTS increased from 4.1 ns (CZTS) to 10.8 ns (CZCTS) after Cd incorporation. As a result, the *V*_OC_ deficit is reduced by more than 100 mV after Cd‐incorporation.^62^ Though Cd substitution may have limited effect on the disordering issue of the absorber materials, suitable Cd incorporation has been proved to enhance the device performance by improving the crystallinity, suppressing the secondary phases (such as ZnS), modifying the doping level, or a combination of them. However, concerning the future large‐scale commercialization, the highly toxic and environmental hazardous nature of Cd could be a potential obstacle.

### 2.3 Ba Substitution for Zn

To control the cation disorder, Xiao et al. used distant atoms such as a group II element barium(Ba) or strontium(Sr), which is far from the group I element (Cu) and group IV element tin(Sn) in the periodical table of elements to replace Zn.^64^ The Ba^2+^(1.56 Å) and Sr^2+^(1.4 Å) may be promising substitutions for Zn^2+^(0.74 Å) to avoid cation disordering because high formation energy of Cu–Ba/Sr and Sn–Ba/Sr antisite defects is expected in Cu_2_BaSn(S,Se)_4_ and Cu_2_SrSn(S,Se)_4_ system, which should not only be attributed to the large mismatch of ionic radius between Ba^2+^ (Sr^2+^) and Cu^+^ (0.74 Å), as well as Ba^2+^ (Sr^2+^) and Sn^4+^ (0.69 Å), but also be attributed to the large difference of electronic properties of Ba and Sr compared to that of Cu and Sn.^14^

**Figure 8:**
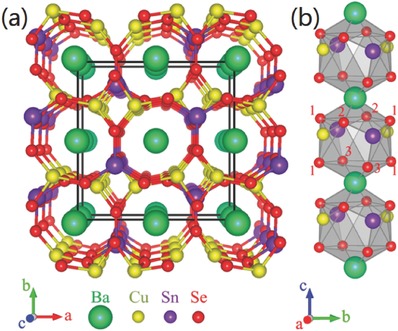
a) Crystal structure of Cu_2_BaSnSe_4_. b) Local symmetryof the cage‐like spaces involved in the tetrahedra channels along[001]. Reproduced with permission.^64^ Copyright 2016, American Chemical Society.

Detailed analysis of the element mutation in I_2_−II−IV−VI_4_ semiconductors by Wang et al. predicts that pure sulfide Cu_2_BaSnS_4_ and Cu_2_SrSnS_4_ kesterite phase will be automatically broken down into binary and ternary compounds because of the phase instability,^39^ which is consistent with the results of Hong et al.^65^ However, Xiao et al. found that chemical potential window that can thermodynamically stabilize the highly symmetrical orthorhombic pure selenide Cu_2_BaSnSe_4_ (CBTSe) is rather large (**Figure** **8**).^64^ Though the bandgap of CBTSe (≈1.72 eV) is not ideal as absorber for single‐junction solar cells according to the S–Q limit, it is an ideal material for the top cell in tandem solar cells and solar‐driven water splitting.^64^ Owing to the large structure difference between tetragonal CZTSSe and orthorhombic Cu_2_BaSn(S,Se)_4_ (CBTSSe), the formation energy of Cu_2_(Zn,Ba)Sn(S,Se)_4_ (CZBTSSe) solid solution is expected to be high. Therefore, rare research has been conducted on CZBTSSe solid solution.

As for the bandgap of Cu_2_BaSnSe*_x_*S_4‐_ *_x_*, a weak indirect gap is observed as 0 ≤*x* ≤ 3, and the value of bandgap is decreased from 2.0–1.6 eV.^41^ The bandgap shows the lowest value of 1.55 eV as *x* = 3, which is the most suitable value for application as absorber material of a single‐junction solar cell.^66^ In contrast, the Cu_2_BaSnSe_4_ shows a direct bandgap of 1.72 eV. Xiao et al. confirmed that the VBM is mainly composed of Se 4p and Cu 3d, and the CBM consist of Sn 5s and Se 4p in the CBTSe.^64^ As for CBTS, Hong et al. proposed that Cu 3d and S 3p contribute to the VBM, and the CBM is determined by Sn 5s and S 3p.^65^ This result is in agreement with the DFT calculation results by Ge et al.^67^ Because the S 3p orbital is lower than that of Se 4p, the valence band edge of CBTS is lower than CBTSe. In addition, the Se—Sn bond length is larger than S—Sn bond, which pulls down the CBM. Therefore the bandgap of CBTS is larger than CBTSe.^65^, ^68^

**Figure 9:**
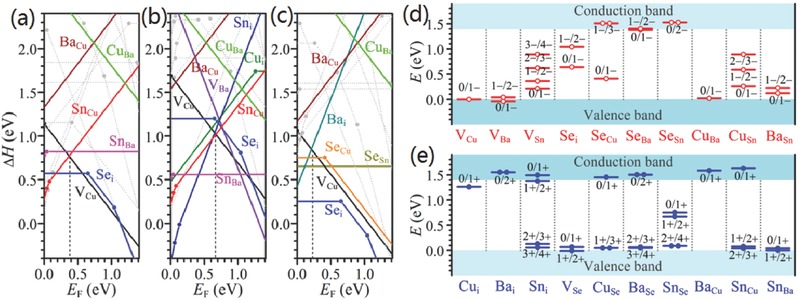
Calculated formation enthalpies of intrinsic defects in CBTSe as a function of Fermi level (*E*_F_) at the chemical potential points a) L (Cu‐poor and Se‐moderate), b) A (Cu‐rich and Se‐poor), and c) U (Cu‐poor and Se‐rich). Calculated charge‐state transition levels of d) intrinsic acceptors and e) intrinsic donors in CBTSe. Reproduced with permission.^64^ Copyright 2016, American Chemical Society.

The calculated defects formation energy and charge‐state transition energy levels of intrinsic point defects in CBTSe system by Xiao et al. are shown in **Figure** **9**.^64^ The good thing is that most of the Ba‐related defects (V_Ba_, Ba_i_, Ba_Cu_, Cu_Ba_, Ba_Se_, Se_Ba_ and Sn_Ba_) are out of the bandgap or very shallow, except that Ba_Sn_ is not a shallow acceptor (about 0.1–0.2 eV higher than the VBM). Moreover, the calculated results show that the formation energies of Ba_Cu_ and Cu_Ba_ anti‐site defects are much higher than those of defects, such as V_Cu_, Se_i_, Cu_i_ etc., no matter in Cu‐poor or Cu‐rich conditions. These results indicates the issue of cation disordering and associated band tailing can be well addressed in CBTSe system and less *V*_OC_ deficit can be expected.

**Figure 10:**
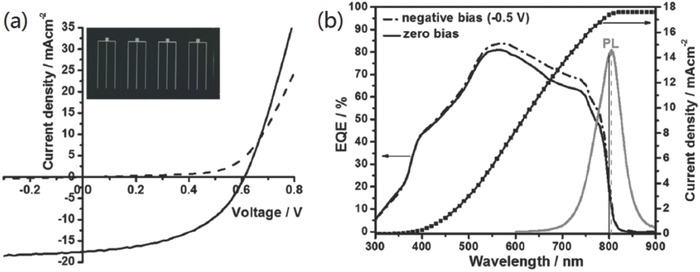
a) Light (solid line) and dark (dashed line) *J–V* characteristics (inset shows a set of four PV devices on glass substrates), and b) EQE spectra of the current champion air‐annealed (200 °C for 3 min) Cu_2_BaSnS_4−_ *_x_*Se*_x_* (*x* = 3) solar cell with (dashed line) and without (solid line) applied −0.5 V bias (for the EQE data). Reproduced with permission.^66^ Copyright 2017, Wiley‐VCH.

The low level of cation disordering and associated band tailing in CBTSSe is well verified by unanimous experimental results that the PL peaks of CBTSSe have no red‐shifting and can be a good estimate of *E*_g_.^64^ For the first time, Shin et al. successfully prepared the Cu_2_BaSnS_4_ (CBTS)‐based thin film on the Mo substrate using RF sputtering and postsulfurization method.^41^ They obtained a 1.62% efficiency device by adopting the typical device structure used for CZTS solar cells: ITO/i‐ZnO/CdS/absorber/Mo/glass. In order to reduce the bandgap close to the optimal range for single‐junction solar cell, they increased the Se content by additional selenization at 570 °C for 5 min after sulfurization. The completed device was annealed at 200 °C for 3 min in air conditions. Finally, the power conversion efficiency reaches to 5.2% (**Figure** **10**a,b).^66^ As indicated by the low EQE response at the short wavelength region, the device may suffer from severe interface recombination, which is probably caused by undesirable conduction band offset at the CdS/CBTSSe interface.

Ge et al. also obtained a 1.57% efficiency Cu_2_BaSn(S_0.83_,Se_0.17_)_4_ solar cell on FTO by sputtering.^68^ By inserting a CdS:O buffer layer with 3% O_2_ content at the junction interface, they significantly improve the *V*_OC_ to 0.9–1.0 V with a bandgap of 2.0 eV. A 2.03% efficiency device was fabricated using a double buffer layer structure: FTO/CBTS/CdS:O/CdS/ZnO/AZO.^69^ Preliminary researches on CBTSSe devices have demonstrated the relatively low level of cation disordering and associated band tailing. Higher performance can be expected with more in‐depth understanding of this family of materials and further device optimization.

### 2.4 Other Transition Metal Substitution

Some other transition metal elements such as Mn, Fe, Co, and Ni are also tried as a substitution for Zn in CZTSSe, due to their analogous structure to CZTSSe, suitable direct bandgap for single‐junction solar cells and associated with high absorption coefficient.^70^, ^71^ Considerable experimental researches have been performed on these materials. However, there are seldom results reported on the device performance by so far. Most of the devices are below 1% efficiency, except a 2.9% efficiency Cu_2_FeSnS_4_/Bi_2_S_3_ device fabricated by Chatterjee et al.^72^ To explore more potential of the Cu_2_MSn(S, Se)_4_ (M = Mn, Fe, Co, Ni)‐based materials, more in‐depth investigation on their thermodynamic stability, electronic structure, and defects properties are needed.

## 3 Optimizing the Bandgap of Absorber and Engineering Its Bandgap Grading

The bandgap of absorber material is another important factor, which has significant influence on the *V*_OC_ deficit and related device performance. Theoretically, the bandgap of CZTSSe and CIGS should be adjusted to about 1.4–1.5 eV, which is the optimal value that expected to achieve highest theoretical efficiency according to the S–Q limit.^28^, ^73^ However, the current practical optimal bandgap of CZTSSe devices is much lower (1.1–1.2 eV).^7^, ^8^, ^74^ CZTSSe solar cells with high S content and large bandgap close to 1.5 eV usually output lower efficiency than the CZTSSe solar cells with small bandgap. One issue should be considered is the degradation of electronic properties of absorber as the bandgap is enlarged by tuning the ratio of cation or anion. For example, the transition energy level of Ga_Cu_ is deeper than that of In_Cu_, which dramatically deteriorates the device performance if the ratio of Ga/(Ga+In) is too high (Ga/(Ga+In) > 0.4).^75^ Analogously, the transition energy level of point defects (including the dominant Cu_Zn_ antisite defects) in CZTS is also deeper than that in CZTSe with small bandgap,^23^ which leads to more nonradiative recombination loss in the bulk and grain boundaries. Another issue that limits the practical optimal bandgap of the CZTSSe absorber is the undesirable CBO at the buffer/absorber interface, especially for the pure sulfide CZTS solar cells.^11^ Despite these challenges, preparing absorber layer with large bandgap (1.3–1.5 eV) and excellent electronic property is a promising direction to explore more potential of kesterite solar cells. Besides, with respect to the engineering of bandgap, another prospective strategy to improve the photovoltaic performance of kesterite solar cells is constructing a graded bandgap similar to the “V” type bandgap grading of CIGS solar cells,^6^, ^8^, ^9^ which is regarded as the optimal solution to balance the trade‐off between *V*_oc_ and *J*_sc_. In this section, we discuss the recent efforts paid to optimize the bandgap of the absorber layer and improve the *V*_OC_ of devices by cation substitution.

### 3.1 Engineering Absorber Bandgap and Conduction Band Offset at Junction Interface

In general, a “spike‐like” CBO (Δ*E*_C_ = *E*_C buffer_ − *E*_C absorber_ > 0) is critical to ensure that the space‐charge‐region (SCR) recombination barrier, *Φ*_b_ is almost equal to the bandgap of absorber material *E*_g_ for ideal absorber semiconductor without any band tailing. *Φ*_b_ is a strong determinant of *V*_OC_ following the equation

(1) $$V_{\mathrm{OC}}=\frac{\Phi_{b}}{q}-\frac{AkT}{q}\ln\left(\frac{J_{00}}{J_{L}}\right)$$

where *q* is the unit charge, *A* is the ideal diode quality factor, *k* is the Boltzmann constant, *T* is the temperature, *J*_00_ is the reverse saturation diode current prefactor and *J*_L_ is the light generated current density.^76^, ^77^, ^78^ In the case of cliff‐like CBO (Δ*E*_C_ < 0), *Φ*_b_ can be significantly smaller than *E*_g_, which inevitably causes severe interface recombination and large *V*_OC_ deficit. On the other hand, over large CBO (Δ*E*_C_) will block the transport of photogenerated electron current, thus leading to the reduced fill factor and short‐ circuit current density (*J*_SC_). The optimal value of CBO is in a small range of 0.1–0.3 eV according to the device simulation results by Gloeckler et al.^79^ Therefore the match of conduction band edge between the buffer layer and the absorber layer plays a vital role to achieve high power conversion efficiency. In the case of CZTSSe, the effective electronic bandgap determined by PL measurements is smaller than the optical bandgap *E*_g_ because of the severe band tailing, especially for the S‐rich CZTSSe with large bandgap.^80^ Therefore, the SCR recombination barrier *Φ*_b_ will be certainly smaller than the optical bandgap *E*_g_, even if there is no interface recombination. In this circumstance, the value of *Φ*_b_ should be close to the electronic bandgap determined by PL measurements. The PL emission energies of pure selenide CZTSe and pure sulfide CZTS are generally in the range of 0.95–0.98 and 1.2–1.35 eV, respectively.^80^, ^81^, ^82^, ^83^ The *Φ*_b_ of CZTSe devices determined by the temperature‐dependent *V*_oc_ measurements is in the range of 0.90–0.97 eV,^81^ which is close to the PL emission energy. However, the *Φ*_b_ of pure sulfide CZTS devices is typically much lower than the PL emission energy.^83^ The energy gap between *Φ*_b_ and PL emission energy can be as large as about 300 meV, as reported by Platzer‐Björkman et al.^83^ This big difference of *Φ*_b_ and PL emission energy of CZTS should be attributed to the undesirable “cliff‐like” conduction band offset between CdS buffer layer and CZTS absorber. Referring to the theoretical calculated bandgap structure, in the case of typical CdS buffer layer is employed, the CBO of CdS/CZTS is negative (−0.09 eV), while the CBO of CdS/CZTSe is positive (+0.26 eV).^22^ Similar conclusion is also confirmed by various experimental results.^84^, ^85^, ^86^, ^87^

One strategy to address this issue is to find an alternative buffer layer, which can match well with the conduction band of large bandgap CZTSSe with high sulfur content. Alternative buffer layer materials with larger bandgap and higher conduction band edge are extensively studied, such as In_2_S_3_, Zn(O,S), (Zn,Sn)O, (Zn,Cd)S.^88^, ^89^, ^90^, ^91^, ^92^, ^93^ Considerable improvements of *V*_oc_ have been obtained by employing (Zn,Sn)O and (Zn,Cd)S buffer layer using atomic layer deposition (ALD) and successive ionic layer adsorption and reaction (SILAR) methods, respectively.^88^, ^89^ However, the *V*_oc_ deficit of CZTS solar cells is still too large, and more in‐depth investigation into the junction interface is needed.

On the other hand, chemical bath deposited (CBD) CdS is still an attractive and prevalent buffer layer, which is essential for the best‐performance devices because of its soft and conformal coverage with acceptable lattice mismatch with the absorber layer, though CdS has the shortcoming of the parasitic absorption of blue light and toxic constituent.^8^, ^15^ In this situation, at present, adjusting the bandgap of absorber layer for better band alignment with buffer layer is an effective way to reduce the *V*_OC_ deficit caused by interface mismatch. To ensure a favorable slightly positive CBO value with CdS buffer layer, the optimal bandgap of CZTSSe is in the range of about 1.3 to 1.4 eV. Because the bandgap of the kesterite‐based absorber is continuously tunable by various methods involving anion and cation substitution, the bandgap of 1.3 to 1.4 eV is achievable in many ways. However, the real challenge is to achieve an absorber material with desirable bandgap without damaging the electronic properties.

To adjust the bandgap, one choice is to tune the ratio of S/(S+Se) in CZTSSe. Lots of efforts have been taken to demonstrate this idea.^48^, ^94^, ^95^, ^96^, ^97^ However, up to now, the best performance kesterite solar cells adopt small bandgap (1.0–1.15 eV) or large bandgap near 1.5 eV.^8^, ^13^, ^15^ Rare result of high‐performance CZTSSe devices with a medium bandgap of 1.2–1.4 eV is reported. One big challenge that this strategy encounters is the high formation energy of CZTSSe materials with a medium ratio of S/(S+Se), i.e., the solid solubility is low when the content of S and Se is close.^11^, ^22^, ^98^ Therefore, high‐sulfur and low‐sulfur phases segregation are often observed in CZTSSe films with a medium ratio of S/(S+Se), thus leading to severe bandgap fluctuation and large *V*_OC_ deficit.^99^, ^100^

Cation substitution is another prospective option to optimize the bandgap of kesterite‐based absorber materials. The influence of cation types on the band edge was studied by Chen et al.^20^, ^55^, ^98^, ^101^ The first‐principle calculations showed that the conduction band minimum of CZTS(Se) is related to the antibonding Sn s and anion p hybrid orbital while the valence band maximum is mainly controlled by hybridization of Cu d and anion p orbitals. Therefore, Ge (IV) for Sn (IV) substitution, and Ag (I) for Cu (I) substitution will adjust the conduction band edge and valence band edge, respectively.

#### 3.1.1 Germanium Substitution

In the Ge alloyed Cu_2_Zn(Sn,Ge)(S,Se)_4_ (CZTGeSSe), Sn is replaced by the smaller Ge atoms, resulting in enhancement of the s–s and s–p level repulsion between Ge and S/Se atoms and the antibonding conduction band minimum is changed.^102^ These results indicate that the bandgaps of CZTS(Se) can be tuned by the substitution of Sn with Ge. Zamulko et al. reported that the bandgaps of the quaternary chalcogenide semiconductors is linearly related to the group‐IV cations change from Si to Ge and then to Sn.^103^ The variation of bandgaps is attributed to the different size of Si, Ge, and Sn. The large cation size weakens the s–s and s–p level repulsion between IV and VI, leading to the decrease of the antibonding conduction‐band minimum as the size of atoms increases. Based on the above results from first‐principle calculations, various studies on Ge substitution have been conducted to investigate the band structure and device performances.Some experimental bandgaps of the Ge‐doped kesterite‐based CZTSSe thin film are listed in **Table** **1**. Wada and co‐workers verified that the *E*_g_ linearly increases from 0.99 eV (pure CZTSe) to 1.35 eV (CZGSe), measured by NIR–vis–UV spectroscopy.^104^ Khadka and Kim found that the bandgap of CZGTS thin films is in the range of 1.51 ± 0.05 to 1.91 ± 0.05 eV), while that of CZGTSe thin films is in the ranges of 1.07 ± 0.05 to 1.44 ± 0.05 eV. The bandgap of the latter is more suitable for the application in single junction solar cells.^105^

**Table 1:** Bandgap (*E*_g_) of CZTGeS and CZTGeSe absorber layers with different Ge/(Ge+Sn) ratio

Material	Method	Ge/(Ge+Sn) [%]	*E*_g_ [eV]	Ref.
CZTGeS	Chemical vapor transport	10–50	1.59–1.94	106
CZTGeS	Spray‐based deposition	0–100	1.51–1.91	42
CZTGSe	Hydrazine processed	0–40	1.08–1.15	107
CZTGSe	Solid solution powders	0–100	0.99–1.35	104
CZTGSe	Spray‐based deposition	0–100	1.07–1.44	42
CZTGSe	Coevaporating	0–100	1.0–1.4	108
CZTGSe	Electrodeposition	40	1.15	109

In past years, many efforts have been taken to investigate the influence of Ge‐incorporation on the device performance of kesterite thin film solar cells.^106^, ^107^, ^108^, ^109^, ^110^ In 2012, Guo et al. obtained an 8.4%‐efficient CZTGeSSe solar cell with Ge/(Ge+Sn) = 0.17 by a nanoparticle based method with Ge contained precursor.^111^ The photovoltaic parameters *V*_oc_, FF, and efficiency of the CZTGeSSe device were significantly improved due to the participation of Ge. Mitzi and co‐workers obtained 9.14% efficiency Cu_2_Zn(Sn_1‐_ *_y_*Ge*_y_*) Se_4_ solar cell with 40% Ge substitution by hydrazine‐based solution process.^107^ Compared to the pure CZTSe device fabricated with an analog process, the bandgap of the 40% Ge‐substituted device is improved from 1.08 to 1.15 eV. Meanwhile, the *V*_OC_ is improved from 0.423 to 0.476 V.^107^ The *V*_oc_ deficit of the Ge incorporated device is 0.674 V, which increases compared with that of the undoped device (0.657 V). Little enhancement of power conversion efficiency (PCE) is obtained by Ge‐substitution in this study, which may be attributed to the Ge loss during high‐temperature processing.

**Figure 11:**
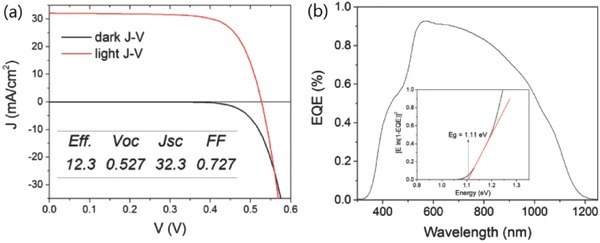
Characterization of the 12.3% efficient CZTGSe thin‐film solarcell: a) *I*–*V* measurement results and b) EQE results. The plot of [*E* ln(1 − EQE)]^2^ vs *E* used to determine the bandgap is shown in the inset of the EQE figure. Reproduced with permission.^113^ Copyright 2016,The Japan Society of Applied Physics.

Despite the bandgap of CZTSe absorber layers are significantly improved by partially substitution of Sn with Ge, Ge changes into volatile GeSe_2_ phases and leads to Ge loss during the high‐temperature selenization process, thus deteriorating the device performance.^107^ By changing the Ge source from GeCl_4_ to GeI_4_, Agrawal and co‐workers reduced the bulk Ge losses significantly and improved the PCE of CZTGeSSe thin‐film solar cells to 9.4%.^112^ Kim et al. prepared the CZTGSe thin films by a co‐evaporation method.^108^ They suppress the Ge and Sn loss successfully by introducing GeSe_2_, SnSe_2_, and Se pellets into the furnace during selenization. The *V*_OC_ of CZTGSe devices increases from 0.44 to 0.58 V as the bandgap is enlarged from 1.1 to 1.25 eV. A 10% efficiency device with *E*_g_ = 1.19 eV, *V*_oc_ = 0.543 V, and *V*_oc_ deficit = 0.647 V was fabricated by this method. Afterwards, Kim et al. further improved the efficiency of CZTGSe device up to 12.3% with *E*_g_ = 1.11 eV, *V*_OC_ = 527 mV, FF = 0.727 by the same method (**Figure** **11**).^113^ The champion CZTGSe device exhibits a greatly improved *V*_OC_ deficit of 0.583 V, which benefits from the reduced band tailing through fine control of the Ge/(Ge+Sn) ratio.

**Figure 12:**
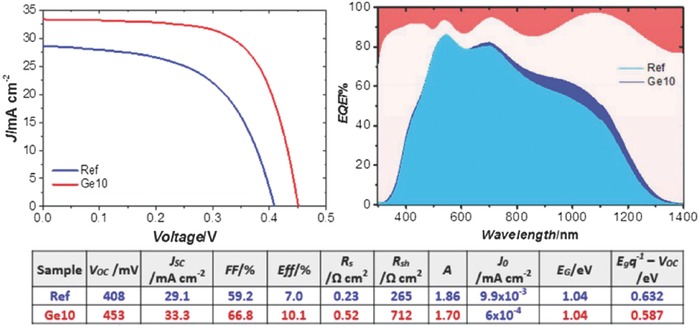
*J*–*V* curves and the corresponding optoelectronic parameters of reference and Ge10 solar cells (left), and EQE plot of both samples (right). In the EQE plot, the upper orange area represents the reflectance of both solar cells. Reproduced with permission.^114^ Copyright 2015, Wiley‐VCH.

Another significant effect of Ge‐incorporation is found by Giraldo et al.^114^ The Ge‐doped CZTSe precursors were prepared by sputtering Cu/Sn/Cu/Zn stacks followed by depositing Ge nanolayers with different thickness by thermal evaporation method. The performance of CZTSe devices was boosted from 7% to 10.1% by only 4% Ge substitution. As shown in **Figure** **12**, The *V*_oc_ of CZTSe thin film solar cells with 10 nm thick Ge layer increases significantly from 408 to 453 mV compared with the reference CZTSe. Consequently, the voltage deficit reduces from 632 to 587 mV. The EQE data and PL spectra indicate that the *E*_g_ of Ge‐doped CZTSe absorber layers is independent of such small amount of Ge substitution. This significant boost of device performance is attributed to the improved crystalline quality of absorber layer because a liquid phase Ge*_x_*Se*_y_*_(l)_ (≈85 at%Se) is formed during selenization, which can effectively assist the growth of CZTSe crystal.^114^ In 2016, Shen and co‐workers also prepared Ge‐doped CZTSSe thin films by a sputtering method.^115^ The authors observed that a small amount of Ge substitution could reduce the pin holes and suppress the bulk recombination of the CZTSSe absorber layer.

Extensive studies on Cu_2_Zn(Sn_1–_ *_x_*Ge*_x_*)Se_4_ thin films were also conducted by other methods, such as electrochemical deposition (ED), pulse laser deposition (PLD), hot injection, and chemical spray pyrolysis.^108^, ^109^, ^116^, ^117^ The bandgap of Cu_2_Zn(Sn_1–_ *_x_*Ge*_x_*)(S,Se)_4_ thin film can be tuned by controlling the ratio of Ge/(Ge+Sn) in the precursors. However, besides the bandgap of the absorber, device performance strongly depends on the absorber fabrication processes. More efforts to better understand the growth mechanism and improve the preparation process of Cu_2_Zn(Sn_1–_ *_x_*Ge*_x_*)(S,Se)_4_ thin film is still in need.

#### 3.1.2 Silver and Cadmium Substitution

The bandgaps of Ag‐substituted (Ag*_x_*Cu_1_ *_x_*)ZnSnSe_4_ (ACZTSe) and (Ag*_x_*Cu_1–_ *_x_*)ZnSnS_4_ (ACZTS) are in range of 1.0 to 1.35 eV and 1.5 to 2.05 eV, respectively, according to theoretical calculation and experimental results.^118^ The sulfide ACZTS with larger bandgap is more suitable as photocatalytic materials,^119^ while the selenide AZTSe with smaller bandgap is more suitable for single junction solar cells concerning the match with solar spectrum.^28^ However, Ag substitution reduces the good p‐type conductivity of absorber and even change it to n‐type when the amount of Ag substitution is high.^55^, ^120^ In this situation, high‐level Ag substitution in CZTSe is not a favorable option if using typical device structure of TCO/CdS/ACTSe/Mo back contact. Thus, studies on Ag‐incorporated CZTSSe solar cells usually adopt low Ag content from 3% to 10%.^38^, ^57^, ^121^, ^122^ Because the bowing of the bandgap of ACZTSe occurs near the region of Ag/(Ag+Cu) = 0.2,^56^, ^122^ the low level of Ag substitution (<10%) has little influence on the bandgap of absorber materials.^57^ The commonly observed *V*_OC_ improvement after slight Ag substitution mainly results from the reduced antisite defects and associated band tailing as discussed in Section 2.1.

Nevertheless, n‐type AZTSe takes advantages of a low level of cation disordering and less band tailing verified by substantially less PL red‐shifting and small Urbach energy observed in several independent researches.^57^, ^123^, ^124^, ^125^ Therefore, n‐type AZTSe could also be a promising absorber material if it is applied in a new configuration with suitable p‐type emitter materials (such as p‐type MoO*_x_*) and corresponding charge transfer layer, which can accommodate the n‐type nature of AZTSe absorber. Recently, Gershon et al. reported a new architecture with a SnO:F/AZTSe/MoO_3_/ITO structure, which demonstrated over 5% efficiency based on their primary researches.^120^ Large photovoltaic potential of AZTSe will be demonstrated soon. To achieve this goal, extensive in‐depth investigation of material processing, band alignment, and interface engineering is needed.

Besides, Cd substitution for Zn is another feasible way to optimize the bandgap of CZTSSe solar cells. Since Cd is in the same group with Zn and its ionic size is larger than that of Zn, the structure of Cd‐substituted materials Cu_2_(Cd*_x_*Zn_1–_ *_x_*)Sn(S, Se)_4_ (CZCTSSe) is similar with CZTSSe. Meanwhile, the bandgap of CZCTSSe is smaller than that of CZTSSe.^37^, ^126^ Pure sulfide quaternary material Cu_2_CdSnS_4_ (CCTS) crystallizes in stannite structure with a bandgap of about 1.4 eV, which is ideal for single‐junction solar cell application.^127^ However, the results of theoretical calculation indicate that the defects structure of CCTS is very similar with CZTS, in which detrimental dominant cation disordering still exists.^128^, ^129^ Su et al. reported that the photovoltaic performance of CCTS is inferior.^37^ The good news is their results show that about 40% Cd substitution for Zn significantly improve the device performance from 5.3% to 9.2%, with bandgap adjusted from 1.54 eV (CZTS) to 1.36 eV (CZCTS). The pronounced increase of device performance is attributed to better crystallinity (which is confirmed by some other results), reduced secondary phases (such as ZnS), and optimized conduction band offset. According to the results of first‐principle calculation, Cd substitution will lower the conduction band edge of CZTS by a maximum value of 0.27 eV,^98^ which can change the heterojunction conduction band offset from the detrimental “cliff‐like” to a favorable slightly “spike‐like.” The optimized CBO could be another reasonable explanation why the *V*_oc_ deficit is reduced by more than 100 mV, even though the problem of band tailing is not solved by Cd substitution.

### 3.2 Optimize the *V*_OC_ and *J*_SC_ Trade‐Off by Bandgap Grading

There is an apparent *V*_OC_ and *J*_SC_ trade‐off in single‐junction solar cells: a light absorber with large bandgap is expected to obtain large *V*_OC_ (because of its large recombination barrier in junction interface) and low *J*_SC_ (because of its narrow spectral response range), and vice versa. The S–Q limit of all kinds of single‐junction solar cells is calculated based on this trade‐off, which reflects the extent of the coupling between the uniform distributed bandgap of absorber materials and the solar spectrum.^28^ The *V*_OC_ is mainly determined by the bandgap of the absorber at the junction interface, i.e., the junction recombination barrier, while the *J*_SC_ is mainly determined by the minimum optical bandgap in the depth profile of absorber. The discrepancy between the junction recombination barrier and the minimum optical bandgap can be enlarged by engineering the depth alignment of the bandgap of the absorber, thus gaining large *V*_OC_ and harvesting more solar light (large *J*_SC_) at the same time. Therefore, another promising strategy to break through the *V*_OC_ limit of CZTSSe solar cell is engineering the bandgap grading of light absorber materials.

**Figure 13:**
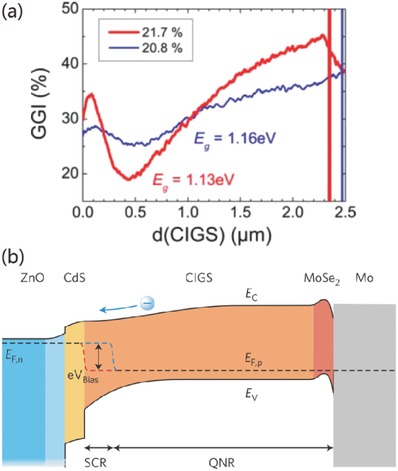
a) Comparison of the GGI grading of the 21.7% (red thick line) and the 20.8% cell (blue thin line) as obtained from SNMS analysis. The vertical red and blue lines represent the end of the CIGS absorber and the transition to the molybdenum back contact. Reproduced with permission.^9^ Copyright 2015, Wiley‐VCH. b) Schematic band diagram of a CIGS solar cell under zero‐bias voltage condition. The Mo back contact is covered with a CIGS absorber layer, which optimally has an average bandgap energy of about 1.2 eV. The p–n junction is formed with a n‐type CdS buffer layer that has a bandgap energy of around 2.4 eV. The front contact consists of a bilayer of intrinsic and aluminum‐doped ZnO layers that have a wide bandgap over 3 eV. Indicated as well are the conduction band energy EC, valence band energy EV, Fermi level EF, space charge region (SCR), and quasi‐neutral region (QNR). Reproduced with permission.^2^ Copyright 2011, Macmillan Publishers Limited. All rights reserved.

The predecessor of kesterite solar cells, CIGSSe solar cell, stands as an excellent example in respect of absorber bandgap grading. As shown in **Figure** **13**a, The “V” type bandgap grading of CIGSe and CIGSSe film has achieved great success by using three‐stage co‐evaporation and the surface sulfurization approaches, respectively.^9^, ^73^, ^130^, ^131^, ^132^ The conduct band minimum (CBM) of CIGSe increases with the content of Ga, while the VBM almost keeps unchanged.^133^ So the conduct band edge of CIGSe can be designed by tuning the ratio of Ga/(Ga+In) along the depth of the film. In the “V” type band structure, high surface bandgap improves the recombination activation energy of the heterojunction, thus leading to high *V*_OC_ (Figure 13b).^2^, ^134^, ^135^, ^136^ The low bandgap at the valley is designed to enlarge the absorption spectrum region of the absorber layer to ensure a large photocurrent; the back gradient of CBM is expected to form a potential field, which facilitates the transport of photogenerated negative charge carriers from the bulk to the front interface. By fine engineering the composition gradient and associated band alignment in CIGS absorber, very high efficiency is obtained even for the devices fabricated on flexible polymer substrates at very low temperature.^2^, ^6^

In a similar way, the gradient of the bandgap of kesterite‐based absorber layer also can be designed by engineering the composition gradient of anion or cation along the depth of the film. The bandgap gradient of absorber layer can be classified into the space charge region (SCR) gradient (namely front gradient), back gradient, and double gradient, which consists of both front and back gradient.^9^, ^137^, ^138^, ^139^, ^140^, ^141^ Theoretically, engineering a graded isoelectronic substitution of any ion in I_2_–II–IV–VI_4_ structure can achieve a graded bandgap. For instance, a substantially graded composition of S/(S+Se), Ge/(Ge+Sn), Ag/(Ag+Cu), or Cd/(Cd+Zn) will surely establishes a graded bandgap. However, the influence on conduction and valence band edges is different, which depends on specific ion substitution. For example, S/Se substitution, Cd/Zn substitution, and Ag/Cu substitution affect both conduction and valence band edges,^20^, ^22^, ^98^, ^128^, ^129^ while Ge/Sn substitution mainly affects the conduction band edge.^42^, ^106^ Graded conduction band edge and valence band edge will establish potential fields or barriers for the transport of electron and hole respectively, which have a strong influence on the collection and separation of photogenerated charge carriers.

Tuning the composition gradient of S/Se along the depth profile of CZTSSe absorber is an attractive method to establish a graded bandgap. Recent research on front band gradient reported by Yang et al. demonstrated a high efficiency of 12.3%, which is the record of vacuum processed CZTSSe solar cells.^142^ In their study, the front bandgap gradient in the depletion region was realized by using SeS_2_/Se hybrid powder source during high‐temperature annealing. *V*_OC_ was improved significantly without *J*_SC_ loss compared to the device with low bandgap. Thus a record *V*_oc_ deficit (576 mV) of kesterite‐based solar cell was obtained. Hwang et al. demonstrated a 10.33% efficiency and the same *V*_oc_ deficit on a CZTSSe solar cell with front graded bandgap.^143^ The front graded bandgap was realized by a single‐step sulfo‐selenization process using Se and H_2_S mixed atmosphere. It needs to be noted that the front graded bandgap by engineering surface S/(S+Se) composition gradient is successful, whereas rare result is reported on CZTSSe film with back graded S/(S+Se) composition. One possible reason is that it is hard to control the replacement speed of Se for S during the selenization process of a CZTS precursor. Owing to the much higher saturated vapor pressure of S vapor than that of Se vapor, the sulfur in CZTS precursor is usually totally replaced by selenium even after a short time selenization process.^12^, ^144^, ^145^

**Figure 14:**
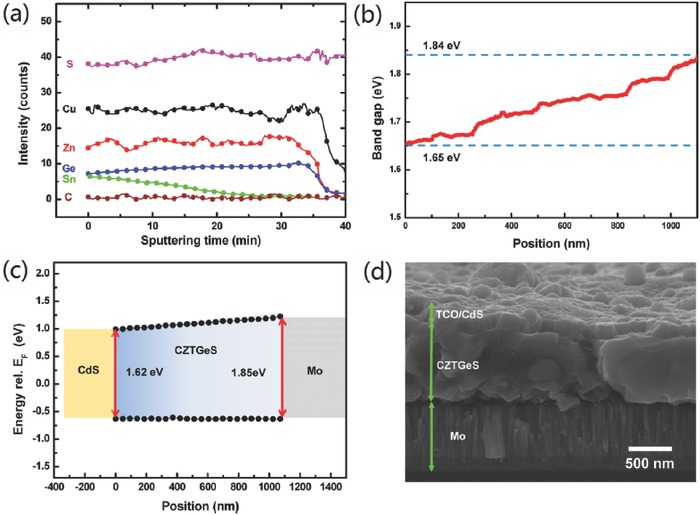
a) SIMS data of bandgap‐graded annealed film to determine the compositional distribution across the film. b) Sn/(Ge+Sn) ratio across the depth of the bandgap‐graded absorber determined by SIMS measurement is converted to the optical bandgap using the linear relationship between the Sn/(Ge+Sn) ratio and optical bandgap. c) Band alignment of graded CZTGeS film measured by UPS and IPES. They measure the VBM and CBM data as a function of position and conversion it to bandgap. The bandgap values were increased from 1.62 to 1.85 eV. d) Bandgap‐graded CZTGeS‐based thin‐film solar cell annealed under AR+H2S (4%) at 530 °C for 30 min. The nanoparticle films were deposited on Mo coated SLG substrate, followed by a successive deposition of CdS, i‐ZnO, and ITO to complete the device fabrication. Reproduced with permission.^146^ Copyright 2014, American Chemical Society .

The large solid solubility of CZTSSe and Cu_2_ZnGe(S, Se)_4_ (CZGeSSe) has been demonstrated by lots of experimental results in which devices with favorable performance were prepared using various components of Ge/(Ge+Sn).^40^, ^42^, ^107^, ^111^, ^146^, ^147^, ^148^, ^149^, ^150^ Therefore, engineering the gradient Ge/(Ge+Sn) composition is a promising method to establish graded bandgap for kesterite absorber layer. Chen et al. found that the bandgap of CZTGSSe can be widely tuned from 0.96 to 2.0 eV, which allows large bandgap gradient in this material.^151^ However, attributed to the fast diffusion of elemental Ge, Sn, liquid Ge*_x_*Se*_y_* and SnSe_2_ intermediate phases, the control of Ge/(Ge+Sn) composition gradient is difficult especially for the absorber films prepared with metallic precursors.^42^, ^44^, ^106^, ^114^ Concerning the diffusion speed of Ge and Sn elements, the precursors consist of CZGeTSSe nanocrystals take advantages over the others because all the elements in nanocrystal‐based precursors are strongly bonded by the other elements. As shown in **Figure** **14**, Kim et al. reported a Ge‐alloyed CZTGeS solar cell with a bandgap gradient from 1.62 eV at the surface increasing to 1.84 eV at the back surface.^146^ The precursor was prepared based on metal chalcogenide complex ligand capped nanocrystals. Compared with ungraded bandgap device, the performance of the bandgap‐graded device was improved from 4.8% to 6.3%. The graded conduction band edge facilitates the collection of photogenerated electrons, which translates to the pronounced increase of EQE response and significant improvement of *J*_SC_. Besides, the *V*_OC_ is also improved even though the effective bandgap is smaller than the device without bandgap grading because of the reduced bulk recombination. Nevertheless, the efficiency of this bandgap‐graded solar cell is still far from the state‐of‐the‐art of kesterite solar cells. Because the Ge‐ incorporated CZTGeS has higher conduction band edge than CZTS, the CZTGeS device must suffer from the severe interface recombination caused by undesirable “cliff‐like” conduction band offset with CdS buffer layer.

Another attractive method to realize a graded bandgap for kesterite solar cells is engineering the gradient composition of Ag/(Ag+Cu). The bandgap of ACZTSe and ACZTS is continuously tunable from 1.0 to 1.35 eV and 1.5 to 2.05 eV, respectively.^118^, ^122^ Therefore, the bandgap span of (Ag, Cu)_2_ZnSn(S, Se)_4_ (ACZTSSe) is as large as from 1.0 to 2.05 eV, which provides a large processing window for the graded band engineering. However, similar to Cu^+^, the diffusion speed of Ag^+^ is also very quick during high‐temperature annealing process.^152^ Moreover, Ag can act as a fluxing agent, which can effectively facilitate the growth of large crystals.^38^, ^57^ This makes it difficult to control the gradient Ag/(Cu+Ag) distribution along the thickness of absorber layer if typical high‐temperature annealing process (above 500 °C) is performed. Fortunately, as observed in several researches, the effect of fluxing agent of Ag can significantly reduce the selenization temperature and enhance the crystallization of ACZTSSe,^60^ which provides a possibility to perform the selenization process in a relatively low temperature under which the diffusion of Ag^+^ and Cu^+^ can be controlled.

**Figure 15:**
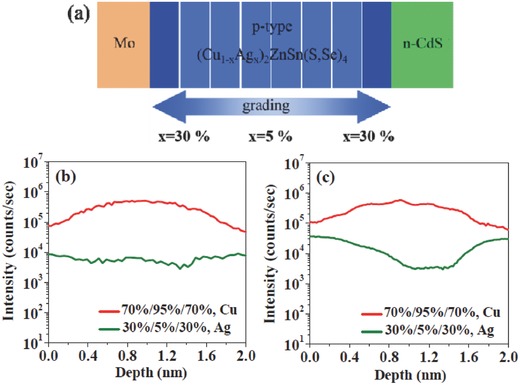
a) Schematic of the Ag‐composition‐graded CAZTSSe device. Note that Cu_Zn_ defects are everywhere inside the CAZTSSe absorber; however, their concentration is extremely high near the CZTSSe/CdS interface because the Fermi level shifts up to a higher level; thus, the formation energy of Cu_Zn_ defects decreases. When a high concentration of Ag is introduced near the interface, the formation of Cu_Zn_ defects is suppressed and Fermi level pinning is retarded. The SIMS depth profiles of the 30%/5%/30% Ag‐graded CAZTSSe samples selenized at b) 550 °C, and c) 480 °C. The scan direction is from the Mo substrate to the CdS interface. Reproduced with permission.^153^ Copyright 2017, The Royal Society of Chemistry.

Recently, Wu et al. reported an 11.2% efficiency double Ag‐graded ACZTSSe solar cell.^153^ They designed a sandwich‐like precursors, which were deposited by a solution based process with high Ag content (20% to 40%) at the bottom and top layer, and low Ag content (5%) in the middle region. The fast diffusion of Ag^+^ is retarded efficiently when the selenization temperature is reduced to 480 °C, and a V‐shape Ag‐gradient is observed, which means a double graded bandgap of ACZTSSe is established for the first time, as shown in **Figure** **15**. The 30%/5%/30% sandwich‐like Ag‐substituted precursor offered the best device performance beyond 11%.^153^ The improvement of device performance mainly arises from the larger band bending and enlarged depletion region width, both of which benefit from the high extent of surface Ag substitution which reduces Cu_Zn_ p‐type defects and provides more weak n‐type defects near the junction interface. This encouraging result opens a new window in engineering gradient bandgap for kesterite thin‐film solar cells.

However, concerning the band alignment between Ag_2_ZnSnS_4_ (AZTS) and Cu_2_ZnSnS_4_ (CZTS), back Ag gradient is not favorable for photogenerated charge collection. According to the results of first principle calculation by Chen et al., a high concentration of Ag‐substitution will lower both of conduction band edge and valence band edge.^55^ So a high concentration of Ag‐substitution at the back interface will generate a reverse potential field driving the photogenerated electrons to the Mo back contact and repelling the photogenerated holes away from the Mo back contact, thus leading to considerable back interface recombination. Even worse, the poor p‐type conductivity of high Ag‐substituted bottom layer will also hinder the collection of hole carrier, leading to large series resistance. On the other hand, a single front Ag‐gradient is appropriate to facilitate the charge separation and collection near the junction interface. The lower conduction band edge originated from high Ag‐substitution at the interface not only avoids conduction band barrier usually observed on CIGS solar cells with strong surface Ga gradient, but also facilitate the electron extraction from the absorber to the front contact. At the same time, the lower valence band edge near the interface will repel the hole carrier away from the junction interface, which can efficiently reduce the detrimental interface recombination. Based on above analysis, more encouraging results can be expected for the Ag‐graded ACZTSSe devices with optimized processing and architecture.

## 4 Engineering the Interface Defects and Band Bending

Besides the abundant bulk defects and associated disordering and band tailing, the defects type at the interface and related pining of Fermi level plays another critical role in the charge transport, separation, and extraction, which have a significant effect on the overall photovoltaic performance including *V*_OC_, *J*_SC_, and fill factor.^34^, ^65^, ^154^, ^155^ For the typical n^+^–p single‐side abrupt junction‐based thin film solar cells without interface defects, the band bending extent of the p‐type absorber layer is mainly determined by the carrier concentration of absorber.^88^, ^156^ In this case, the interface Fermi level locates close to the CBM of the absorber layer. However, the interface defects are inevitable and play an essential role in device operation in practice.^157^, ^158^, ^159^

**Figure 16:**
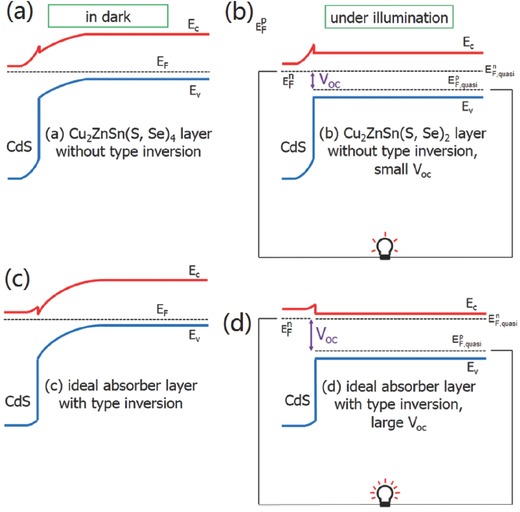
The band diagram of solar cells in the dark (left) and under illumination (right): a) the CdS/Cu_2_ZnSn(S,Se)_4_ solar cell with no type inversion (the Fermi level near the interface is pinned at the middle of the bandgap) and small band bending in the absorber layer, b) the CdS/Cu_2_ZnSn(S,Se)_4_ solar cell with a small *V*_oc_ under the flat band condition, c) an ideal absorber layer with type inversion (the Fermi level near the interface is close to the conduction band) and a large band bending, and d) an ideal absorber layer with a large *V*_oc_ under the fl at band condition. The band bending in the n‐type CdS layer is not changed much under illumination because its absorption of visible light is weak and the concentration of the majority of electron carriers is not changed significantly by the photogenerated carriers. As we can see, the maximum Voc under illumination is derived mostly from the band bending of the absorber layer in the dark. Reproduced with permission.^55^ Copyright 2015, Wiley‐VCH.

As a terminal of periodical microcrystals which constitute the absorber layer, the surface usually has different chemical composition from the bulk, which consequently constructs a different defects structure on the surface of the absorber layer. For example, a Cu‐depleted surface is commonly observed in generally adopted Cu‐poor CIGS films.^135^, ^160^, ^161^ This Cu‐depleted surface usually consists of Cu‐poor ternary phases, such as CuIn_3_Se_5_, CuIn_5_Se_8_ (OVC phases), in which the dominant defect is donor‐like In_Cu_.^162^ Moreover, the large population of Cu vacancy on the Cu‐depleted CIGS surface is much likely to be occupied by Cd^2+^ during the CBD deposition of CdS buffer layer, possibly forming a large population of Cd_Cu_ donor‐like defects at the junction interface.^160^ These donor‐like defects at the surface lead to surface‐type inversion. Moreover, the surface Fermi level of CIGS film will be pinned at the transition energy level of dominant donor‐like defects, which is close to the conduction band edge if the defect density is high enough.^79^, ^154^, ^163^ In this case, a large band bending of absorber layer spontaneously forms at the junction interface. This strong band bending will generate larger built‐in potential, stronger built‐in electric field, and wider space charge region, i.e., a substantially stronger junction, which can be translated to larger Fermi level splitting at the thermodynamic equilibrium under illumination, a stronger driving force for charge separation at the junction, and higher photogenerated charge collection efficiency. Consequently, the *V*oc, fill factor, and short‐circuit current density (*J*_SC_) will be improved significantly. Moreover, the surface type inversion in CIGS solar cell changes the heterojunction structure into a shallow buried homojunction, which is directly evidenced by electron beam introduced current (EBIC) spectra.^7^, ^164^ As a consequence, usually observed severe interface recombination at heterojunction interfaces caused by lattice mismatch and dangling bonds can be avoided. On the contrary, if the surface Fermi level of a p‐type absorber is pinned near the valence band edge by large population of acceptor‐like defects, the band bending of absorber layer will be dramatically decreased, and the junction is relatively weak. The different band diagrams of these two distinct heterojunctions in dark with zero‐bias and under illumination under the flat band condition (operating at bias close to *V*_OC_) are shown in **Figure** **16**, 55

Unfortunately, the conduction band edge of CZTS is much higher than its pining energy of the Fermi level for n‐type doping,^11^, ^22^ which means it is difficult to obtain an n‐type CZTS material neither by intrinsic defects doping, nor by extrinsic doping.^22^ While the conduction band edge of pure selenide CZTSe is close to its n‐type doping pining energy, indicating it is possible to get an n‐type CZTSe by intrinsic or extrinsic doping.^11^, ^22^ Several experimental results have reported n‐type CZTSe materials under a Sn‐rich and Zn‐poor condition.^165^, ^166^ In these materials, the dominant donor‐like defects should be Sn_Zn_ antisites, and a large population of 2Cu_Zn_+Sn_Zn_ clusters should exist. Therefore, by engineering a Sn‐rich and Zn‐poor surface, surface‐type inversion could be achieved theoretically. However, the Sn_Zn_ antisites are deep level defects, and 2Cu_Zn_+Sn_Zn_ clusters are also deep trap states which may reduce the bandgap and act as recombination center. These results explain the poor *V*_OC_ and severe band tailing of Sn‐rich and Zn‐poor kesterite solar cells.^45^ In a word, engineering a Sn‐rich and Zn‐poor surface to realize surface type inversion of the CZTSSe absorber layer is not a favorable option.

It can be expected that CZTSSe material with relatively Cu‐poor and Zn‐rich composition will be dominated by donor‐like Zn_Cu_ defects and exhibit an n‐type conductivity according to the chemical potential dependent defects formation energy. However, up to now, the experimental result of n‐type CZTSSe material under Zn‐rich condition is rare. Our previous work reveals that CZTSSe with relatively Cu‐poor and Zn‐rich composition (Cu/Sn) ≈ 1.70, Zn/Sn ≈ 1.30) exhibits much stronger p‐type conductivity than that with slightly Cu‐poor and Zn‐rich composition.^45^ Meanwhile, n‐type secondary phases Zn(S, Se) is easy to form both at the front surface and at the bottom in this composition.^45^, ^167^, ^168^, ^169^ Theoretically, the n‐type surface Zn(S, Se) phases may enlarge the band bending of the CZTSSe absorber, and surely improve the *V*_OC_. Some experimental results confirm this deduction that a small quantity of surface Zn(S, Se) phase is beneficial to improve the *V*_OC_ of CZTSSe devices.^45^, ^170^ It is worth to note that no potential barrier will hinder the electron transfer from the bulk to front contact, because the OVC phases have a similar band structure with CIGS.^171^ However, Zn(S, Se) secondary phases have much larger bandgap and much higher conduction band edge than CZTSSe.^98^ Based on the above‐mentioned results, the large potential barrier arising from the high conduction band edge of Zn(S, Se) phase will severely block the transfer of electron current form bulk to front current, thus dramatically deteriorating the fill factor and *J*_SC,_ and even leading to a kink of current–voltage curves.^45^

Therefore, to achieve surface type inversion and enhance the band bending, an n‐type conductivity phase with similar band structure with CZTSSe is urgently needed. The new emerging kesterite Ag_2_ZnSnSe_4_ (AZTSe) is an ideal candidate as proposed by Chen and co‐workers.^55^ AZTSe is an intrinsic defect doped n‐type semiconductor with a bandgap of about 1.35 eV.^60^ The AZTSe and CZTSSe have similar crystal structure, and the (Ag,Cu)_2_ZnSn(S,Se)_4_ (ACZTSSe) alloys have large solid solubility.^56^ As the Ag‐substitution increases, the p‐type conductivity of ACZTSSe reduces and even changes to n‐type when the content of Ag is high enough.^57^ By engineering an Ag‐graded ACZTSSe absorber with decreased Ag‐substitution form surface to bulk, a p‐type absorber with surface type inversion can be constructed, the interface Fermi level can be pinned close to the CBM, and the band bending of the absorber can be enhanced.^55^ Moreover, Ag substitution for Cu mainly lowers the valence band edge, while the conduction band edge is just lowered slightly, which is very similar to the OVC phases in CIGS system. The favorable band structure of Ag‐graded surface will facilitate the extraction of negative charge carriers and repelled the positive charge carriers away from the interface, i.e., enhancing the separation of electron–hole pairs and reducing interface recombination. Combined with the desirable effect of a graded surface bandgap, enhanced band bending in ACZTSSe absorbers with a Ag‐graded surface is expected to realize a substantial progress in overcoming the *V*_oc_ limit of kesterite‐based solar cells. Most recent research by Wu et al. has observed an enhanced band bending of absorber layer by introducing a surface Ag‐graded composition.^153^ The analysis of ultraviolet photoelectron spectroscopy (UPS) measurements show that the distance between Fermi level and the conduction band of Ag graded CdS/CAZTSSe heterojunction is 100 mV smaller than that of the CdS/CZTSSe heterojunction with a comparable bandgap. This result indicates the Fermi level pinning is weakened. As a consequence, the V_OC_ is improved significantly. Further improvements can be expected with the more delicate engineering of surface Ag‐gradient.

## 5 Summary and Prospective

In summary, this review provides an overview of recent advances of the efforts to break through the *V*_OC_ limit of low cost and environment‐friendly kesterite‐based quaternary chalcogenide solar cells, with a particular focus on the strategies based on cation substitution. Though the kesterite thin‐film solar cells have experienced a rapid progress in the last decades, there is still a long journey to catch up with its predecessors CIGS and CdTe solar cells which have demonstrated more than 20% PCE. The main obstacle in the way is unanimously attributed as the large *V*_OC_ deficit (generally > 0.6 V), which is now commonly attributed to the band and potential fluctuation caused by the substantial band tailing, as verified by commonly observed PL redshifting. The prevailing Cu_Zn_ and Zn_Cu_ antisite defects and associated disordering with relatively low formation energy are recognized as the primary culprit that accounts for the band tailing and related large *V*_OC_ deficit. Besides the cation disordering and associated band tailing, another issue needs to be addressed is the nonoptimized band alignment at the junction interface and the absorber bandgap in order to reduce *V*_oc_ deficit. It is necessary to control over a delicate balance among the absorber bandgap matching with solar spectrum, the bandgap‐related electronic properties, and the conduction band offset at the junction interface. One more critical issue may be responsible for the large *V*_OC_ deficit is the pinning of interface Fermi level to a low energy level by a large population of Cu_Zn_ acceptor‐like defects, which reduces the band bending in the absorber and produces a weak junction, deteriorating both *V*_OC_ and fill factor.

In this review, we mainly consider the promising strategies to address the above‐mentioned issues, including: (i) suppressing the cation disordering by isoelectronic cation substitution, which introduces large ionic size and electronic property mismatch; (ii) optimizing the interface conduction band offset and constructing a graded bandgap by suitable cation substitution; (iii) engineering junction interface defects and enhancing related band bending in the absorber. Concerning the origin of the prevalent Cu–Zn disordering, which caused by almost the same ionic size and similar electronic properties, isoelectronic cation substitution involving large mismatch of ionic size and electronic properties is a feasible way to suppress cation disordering. Ag, Cd, and Ge substitution for Cu, Zn, and Sn, respectively, have been extensively studied. Owing to the large difference of ionic size between Ag and Zn, and the relatively low valence band edge of AZTSSe, the formation energy of Ag_Zn_ and Zn_Ag_ antisite defects is substantially higher than that of Cu_Zn_ and Zn_Cu_. Thus the cation disordering in Ag‐substituted ACZTSSe can be efficiently suppressed. Sharper PL emission peak and much less PL red shifting is observed in ACZTSSe devices by various experimental results, which confirms the theoretical prediction. Meanwhile, relatively high device performance has been achieved based on ACZTSSe materials. Though Cd and Ge substitutions are expected with little effect on suppressing the cation disorder and associated with band tailing according to theoretical calculation, pronounced enhancement of device performance is observed in the Cd, or Ge substituted devices, which is mainly attributed to the improved crystallization and reduced secondary phases. The most recently reported Cu_2_BaSn(S, Se)_4_ materials adopting a very different crystal structure from kesterite is also a promising candidate for improving cation ordering. The reduced band tailing of Cu_2_BaSn(S, Se)_4_ was demonstrated by several experimental results, and show a 5.2% efficiency, which suggests the potential of Cu_2_BaSn(S, Se)_4_ materials as a promising low‐cost and environment‐friendly PV absorber.

Learning from the predecessor CIGS solar cells, modifying the conduction band offset at junction interface is critical to reduce the interface recombination and the associated *V*_oc_‐deficit. By suitable cation or anion substitution, such as Ag/Cu, Cd/Zn, Ge/Sn, S/Se, the bandgap and the conduction band offset of kesterite absorber can be continuously tuned. Among these possibilities, Ge substitution and Cd substitution have demonstrated considerable improvement of device performance by optimizing the bandgap of the absorber. However, systematic investigation on the relation among the absorber bandgap, conduction band offset, and *V*_OC_ deficit is still urgently in need. In addition, engineering a V shape graded absorber band alignment is an attractive strategy to further improve the *V*_OC_ without *J*_SC_ losses. By a front graded S/(S+Se) composition, 12.3% efficiency is achieved with a relatively low *V*_oc_ deficit (576 mV) based on sputtering related processes. Nevertheless, back graded S/(S+Se) composition is a big challenge because the replacement of S with Se during selenization process is too fast to control. On ther other hand, a single back graded band alignment is demonstrated by a graded Ge(Ge+Sn) composition, which effectively improves the collection efficiency of negative charge carriers and also significantly enhances the *V*_OC_ of devices. Very recently, a V shape double‐graded bandgap alignment is realized by front and back gradient of Ag/(Ag+Cu) composition. This new architecture takes apparent advantages of both large *V*_oc_ and large *J*_SC_ compared with the devices with uniform bandgap alignment, resulting in an 11.2% PCE of Ag substituted kesterite device. A big challenge encountered in engineering graded band alignment using isoelectronic cation substitution is the fast cation diffusion speed during high‐temperature annealing, which is inevitable for the growth of absorber film. To tackle this dilemma, the nanoparticle precursor based approaches are more feasible to engineer a graded band alignment than the metallic precursor based approaches.

Besides, engineering the interface defects and enhancing the band bending in the absorber are also promising strategies to overcome the large *V*_OC_ deficit of kesterite solar cells. Unlike the surface of CIGS film which generally exhibits a type inversion originated from the n‐type OVC phases, the surface of CZTSSe is usually dominated by acceptor‐like Cu_Zn_ defects, which pins the interface Fermi level to a low energy level close to the VBM of the absorber, thus weakening the band bending in the absorber layer. Ag‐based kesterite materials Ag_2_ZnSn(S, Se)_4_ are intrinsic n‐type semiconductors with similar crystal and band structure with CZTSSe. Therefore, a high extent surface Ag substitution is a feasible way to achieve a surface type inversion and enhance the band bending in absorber layer, without substantial change in surface band structure. The large photovoltaic potential of front Ag‐graded kesterite‐based device is waiting for exhaustive exploration.

## Conflict of Interest

The authors declare no conflict of interest.
